# The Natural Progress of Disease

**Published:** 1864-06

**Authors:** John Hughes Bennet


### ART. IV.—*The Natural Progress of Disease.* By JOHN HUGHES BENNET, M. D., Edin., F. R. D. E.

It may be laid down as a general law, that diseases are seldom stationary, and that their tendency is to get better or to get worse. While many disorders, from their trifling character, or in consequence of being well known, are at once recognized as capable of disappearing spontaneously, others have been supposed actually to have a destructive or injurious tendency, or to be necessarily fatal. Now the study of disease in modern times has led to a great change in our views on these heads. For example, it was formerly supposed that acute inflammations had, for the most part, a destructive tendency; that suppuration was a great evil, and always required the interference of the surgeon, because an abscess, if so deep-seated that it could not be reached with the knife, seldom got well, and if it burst into an internal cavity, caused death. Again, if inflammation visited the skin, the mucous or serous membranes, or the internal organs, the great object was to prevent it spreading by using the most violent remedies, such as bloodletting, purging, antimony, and low diet, which received the name of antiphlogistics. On the other hand, a tubercular disease, especially when it attacked the lung, was supposed to be almost uniformly fatal, and altogether beyond the reach of art.

Now these conclusions are erroneous. We have previously seen that an analeptic treatment frequently cures tubercular diseases; while the antiphlogistic treatment, formerly supposed capable of cutting short inflammations, not only fails to do so, but constitutes a most fatal practice. Much of this error depended on unacquaintance with the natural progress of disease. Most diseases in vigorous constitutions, so far from having a tendency to destroy, have a marked tendency to get well of themselves; whilst instead of loss of blood, weakness, and prostration being remedies, they are the sources of danger and the chief causes of the fatal result.

Again, malignant growths were supposed to be seated in the blood—an idea which rendered operating useless; whilst innocent growths were supposed to be capable of going away of themselves, or to be the only ones admitting of surgical interference. In this, also, a great change in opinion has been effected; so that cancers like other growths, are now known to have been successfully extirpated.

But further, how is it possible to know the effect of any remedy whatever, unless it be ascertained, in the first instance, not only what is the natural termination, but also the natural duration of a disease? We know that small-pox, scarlatina, measles, and similar affections, run a certain course, and no one thinks of cutting them short, or proposes different kinds of remedies for that purpose. The real principle of treatment is to conduct them to a favorable termination. Should not the same rule apply to many other diseases?

Some years ago Dr. Hamilton Bell stated that fifteen drops of the tincture of muriate of iron was a valuable remedy in erysipelas, but *how* valuable was not shown, because it was not attempted to be proved that the remedy diminished the mortality, or shortened the progress of the disease. Notwithstanding, this remedy was at once largely given, and it was said, with universally good results. I remember accompanying M. Louis, many years ago, in his visit in the Hotel Dieu, and was much struck by seeing many cases there of severe erysipelas of the scalp. On asking him what treatment he pursued, he answered, none at all, because they all rapidly got well of themselves in healthy constitutions. And, in fact, on following these cases from day to day I found that they all did so get well. I need scarcely say that in the Royal Infirmary I have seen many severe cases of erysipelas. I have never given the tincture of muriate of iron, or anything but good diet, with lotions of acetate of lead, flour or oil locally to alleviate irritation, and I have not had a fatal case. Nor has it ever appeared to me that the tincture of muriate of iron could have shortened the progress of the disease. I need scarcely say that any remedy might easily obtain a great reputation if given in diseases that almost always get well of themselves.

Again, look at rheumatism. Every drug and every system of treatment has been tried. In acute cases, bleeding, purging, antimony, mercury, the whole class of sedatives and narcotics, stimulants, quinine and lemon-juice, large doses of alkalies, numerous specifics, hot baths, cold baths, dry frictions and moist applications in every form. Yet under every one of these remedies, however opposite in their nature, notable cures have been performed. Is not the conclusion obvious, that the disease follows a certain progress, and that although many of these remedies may retard convalescence, it has yet to be proved which, if any, shorten its duration, even one hour?

One method of prosecuting therapeutics, therefore, is to investigate—1st, how long a disease naturally takes to get well of itself under favorable circumstances; 2ndly, what is its progress under unfavorable circumstances; and lastly, this being known, how far remedies are capable of shortening its duration. If every young practitioner would dedicate his life to the elucidation of the natural progress of only one disease, he would do more for medical practice than has been accomplished by centuries of empirical trials of remedies.

